# Editorial: Advanced genetic and genomic methods and applications for malaria surveillance

**DOI:** 10.3389/fgene.2026.2000967

**Published:** 2026-10-15

**Authors:** Shazia Ruybal-Pesántez, Johanna H. Kattenberg, Anita Ghansah, Sarah Auburn

**Affiliations:** 1 Barcelona Institute for Global Health, Barcelona, Spain; 2 Instituto de Microbiología, Universidad San Francisco de Quito, Quito, Ecuador; 3 MRC Centre for Global Infectious Disease Analysis, Department of Infectious Disease Epidemiology, Imperial College London, London, United Kingdom; 4 Department of Biomedical Sciences, Institute of Tropical Medicine, Antwerp, Belgium; 5 Department of Parasitology, Noguchi Memorial Institute for Medical Research, University of Ghana, Accra, Ghana; 6 Menzies School of Health Research, Darwin, NT, Australia

**Keywords:** anopheles, drug resistance, genomic epidemiology, importation, malaria molecular surveillance, plasmodium, transmission dynamics

## Introduction

Malaria molecular surveillance (MMS) has advanced rapidly from proof-of-concept to a recognised pillar of the World Health Organization’s strategy for malaria control and elimination. Parasite genotyping now underpins a growing number of surveillance ‘use cases’, including monitoring antimalarial drug resistance, that align with the programmatic questions that national malaria control and elimination programmes (NMCEPs) must answer at different stages of the elimination pathway. High-throughput and, increasingly, targeted and portable sequencing now make it feasible to genotype parasites at the scale that NMCEPs require, while a maturing analytical toolkit translates raw sequence into epidemiologically meaningful signals. However, persistent challenges remain: ensuring that these methods are accessible, appropriately matched to the question at hand, and able to deliver actionable information to programme officers.

This Research Topic was convened to highlight recent innovations and applications of genomics that address the practical questions MMS systems must answer. The articles in this Research Topic collectively demonstrate how genomics is reshaping malaria surveillance, strengthening understanding of parasite biology, and informing control and elimination strategies. Three key themes arise: (i) expanding the genomic toolkit through development of accessible, cost-effective, and increasingly portable genotyping methods spanning both parasite and vector; (ii) translation of those methods into programmatically actionable outputs: resolving transmission dynamics and parasite connectivity on the one hand, and tracking the emergence and spread of antimalarial drug resistance on the other; and (iii) remaining gaps that limit the programmatic integration of genomic data, ranging from accurately detecting imported infections to the persistent under-representation of *Plasmodium vivax* and other non-falciparum species.

Here we summarise the contributions and reflect on where the field must go next.

## Expanding the genomic toolkit

A recurring obstacle to establishing MMS is the cost, infrastructure, and specialist expertise that next-generation sequencing demands, which are not always attainable in the settings where surveillance is most needed. Several contributions address this by developing and validating targeted approaches that recover comparable information to whole genome sequencing (WGS) at substantially lower cost. Niaré et al. present a highly multiplexed molecular inversion probe panel for *P. falciparum* that targets common single-nucleotide polymorphisms (SNPs) and approximates WGS-based assessments of selection and relatedness, bringing genome-scale inference within reach of programmatic budgets. In a complementary evaluation, Cabrera-Sosa et al. benchmark newly developed SNP barcode panels for *P. falciparum* and *P. vivax* against established microsatellite markers in the Peruvian Amazon, providing evidence for panel recommendations. Extending accessibility further still, Adegbola et al. apply portable Oxford Nanopore (ONT) sequencing to characterise drug-resistance loci within an antenatal-care population in Nigeria, illustrating how decentralised platforms can lower the barrier to in-country genotyping. In the context of vector genomics, Boddé et al. demonstrate improved species assignment across the entire *Anopheles* genus that is made possible by applying a targeted-sequencing assay for mosquito genotyping with updated reference databases.

## Understanding parasite diversity and transmission patterns

Genetic and genomic data have the potential to inform on the diversity of parasites and how transmission changes over time and space to monitor the efficacy of existing interventions and guide new strategies. Pereus et al. evaluate *P. falciparum* diversity across mainland Tanzania, using genetic metrics of complexity of infection, parasite connectivity, and transmission intensity, revealing heterogeneity that conventional indicators can miss. Similarly, Fola et al. utilized population genetic metrics to track *P. vivax* transmission indicators through a period of transmission decline and rebound in two endemic areas of Papua New Guinea, showing that genomic signals can capture changing transmission dynamics where they are hardest to measure.

## Monitoring antimalarial resistance

The emergence and spread of antimalarial drug resistance remains one of the greatest threats to malaria control globally. A key use case for parasite genetic data is to detect and track the spread of antimalarial resistance. Kattenberg et al. use genetic surveillance to document the spread of artemisinin-combination-therapy resistance during a period of malaria decline in Vietnam between 2018 and 2020, providing outputs with immediate implications for treatment guidelines and containment. The ONT-based profiling of *crt, dhfr*, and *dhps* markers in pregnant women reported by Adegbola et al. points to new approaches to embed resistance genotyping into existing clinical touchpoints such as antenatal care.

## Gaps and future directions

Collectively, these studies showcase the increasing potential for MMS to inform public health decision-making. Yet real gaps remain between capability and routine use. Safarpour et al. synthesize this challenge for a critical elimination use case, distinguishing imported from locally acquired infections. They find that current methods are still limited by cost, an inability to reliably discriminate between imported and local parasites, challenges in tracking parasite movement in cross-border settings, within-country or at smaller spatial scales, and reliance on cross-sectional summary statistics that only capture snapshots of transmission. Their review also points to a gap in *P. vivax* and other non-falciparum species. Whilst *P. falciparum* accounts for the major burden of global malaria cases, malaria eradication will require concerted efforts to address the unique challenges of the non-falciparum species.

Encouragingly, several contributions reflect the expanding sequencing and bioinformatics capacity, along with growing scientific leadership, within malaria-endemic regions. Investments in regional sequencing hubs, local expertise, and collaborative research networks are enabling countries to generate and interpret genomic data relevant to their own public health priorities. This momentum must be sustained if MMS is to become routine public health practice, rather than be constrained to research. Importantly, the next phase of MMS should focus on the generation of fit-for-purpose data, at the scale and timeliness necessary for translation into decision-making. This will require sustained investment in local infrastructure and capacity, harmonised and tailored analytical frameworks, and closer engagement between genomic scientists and NMCEPs. It will also be critical to better integrate parasite and vector data, as well as molecular, epidemiological, clinical and environmental information into a coherent picture of malaria transmission. As NMCEPs confront evolving biological and operational challenges, genetic data will continue to play an increasingly central role in guiding interventions, informing policy and sustaining progress toward elimination. We hope this Research Topic stimulates further innovation, collaboration, and investment in MMS to accelerate global malaria control and elimination.

